# Radiofrequency Ablation Of Typical Atrial Flutter Via Right Subclavian/jugular Vein Access In A Patient With Implanted Filter In The Inferior Vena Cava

**Published:** 2009-07-01

**Authors:** Jorg Kynast, Panagiotis Margos, Gert Richardt

**Affiliations:** Herzzentrum Bad Segeberg, Bad Segeberg, Germany

**Keywords:** Atrial flutter, Ablation, Superior Vena  Caval Approach

## Abstract

Radiofrequency ablation of Cavotricuspid Isthmus-dependent Atrial Flutter (CTI AFL), a usual and safe therapeutic procedure in interventional electrophysiology with a high success rate, aiming to induce permanent block of conduction over CTI, is normally performed via the femoral access, which allows practical access to the CTI through the inferior vena cava (IVC). In rare cases of obstruction of IVC, ablation of CTI can be performed only through the superior vena cava (SVC) access.  We present a case  of typical atrial flutter that was ablated through the right subclavian/jugular veins because of  iatrogenic obstruction of the IVC due to a previously implanted thrombus  filter. Furthermore we discuss about how we resolved access-related problems of instability during catheter ablation on CTI.

## Introduction

Radiofrequency ablation of Cavotricuspid Isthmus-dependent Atrial Flutter (CTI AFL) is a usual and safe therapeutic procedure in interventional electrophysiology with a high success rate [1-5]. Its aim is to perform an ablation line on the well recognized CTI area of slow conduction to induce permanent block of conduction over this part of the macro-reentry circuit. Normally the procedure is performed via the femoral access, which allows practical access to the CTI through the inferior vena cava.

In rare cases of congenital or iatrogenic obstruction of IVC or bilateral femoral vein thrombosis, ablation of CTI can be performed only via the superior vena cava (SVC) access. We present a case of a patient with typical atrial flutter that was ablated through the right subclavian/jugular veins due to iatrogenic obstruction of the IVC by a previously implanted thrombus  filter.

## Case report

**Figure 1:**
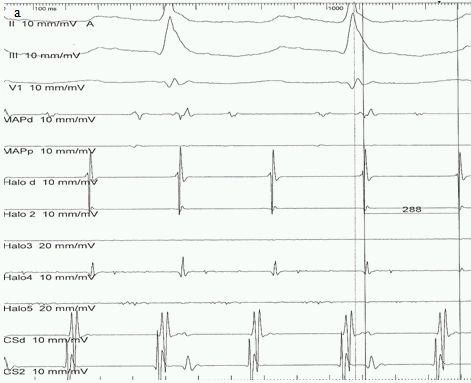
Intracardial signals of AFL with cycle length 288 ms

A 55 year-old woman was referred to our Heart Center due to AFL with fast ventricular response despite medical therapy with verapamil plus beta-blocker, causing palpitations and fatigue on moderate excercise. The patient had a history of recurrent pulmonary embolism; therefore she had been treated in another hospital with bilateral pulmonary thromb-endarterectomy, reconstruction of tricuspid valve by annuloraphy including atriotomy and implantation of an IVC filter (type "Cordis Easy Trap").  The  patient's resting ECG revealed saw tooth pattern in leads II, III and AVF, providing the reasonable diagnosis of typical AFL. Due to the highly symptomatic medically-uncontrolled fast ventricular response of AFL, ablation of CTI was decided and a subclavian/jugular vein access was chosen, as the presence of IVC filter made the femoral vein access impossible. After placing two 6F-sheaths in the right jugular vein and one 8F-sheath in the right subclavian vein, a 4 pole fix-curved and a 10-pole steerable diagnostic catheter were advanced through the jugular sheaths into the Coronary Sinus (CS) and the right atrium (RA). The intracardial signals were compatible with typical counterclockwise AFL (Figure 1) and positive concealed entrainment proved the flutter's isthmus dependence.

**Figure 2:**
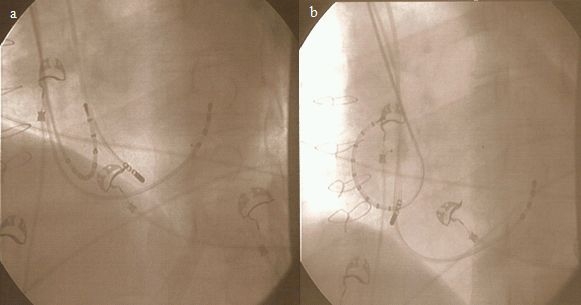
a. LAO 40º: Position of diagnostic 4 Pole catheter in CS, 10 Pole catheter in RA (Halo), Map / Ablation-catheter on CTI. Ablation of ventricular aspect of the CTI. b. Ablation of the atrial aspect of the CTI with the tip of the ablation turned down towards IVC

Subsequently, a 4-pole 8mm-tip ablation catheter was advanced over the 8 F subclavian sheath to the ventricular margin of the CTI zone (Figure 2a). The ablation procedure was started (50W energy, 70ºC temperature-limit, applications of 2-minute duration) and was continued with steady retraction after each application. The initially stable contact of the ablation catheter on the target CTI-zone was lost at the atrial aspect of the CTI; so the ablation line was completed by turning the tip of the ablation catheter into the IVC and re-starting the line from its IVC margin (Figure 2b).

**Figure 3:**
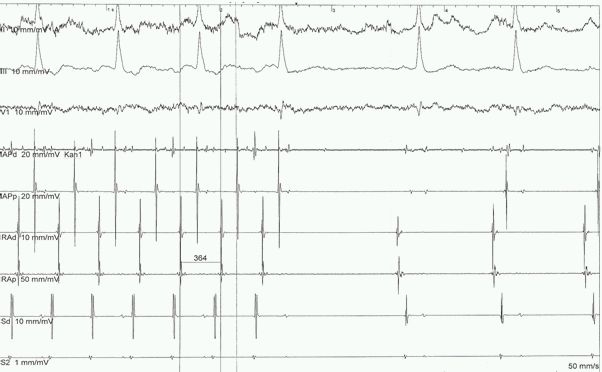
Prolongation of cycle length (364 ms) and conversion into sinus rhythm

By  connecting the two described semi-lines (8 applications, total ablation time 14 min. 35 sec.) we obtained the conversion of AFL to sinus rhythm (Figure 3). Subsequent pacing manoeuvres over the diagnostic catheters confirmed the bidirectional block through the CTI zone. The post-intervention clinical course was uncomplicated and on 3-month follow-up the patient was asymptomatic in sinus rhythm, on oral anticoagulation and betablockers.

## Discussion

For ablation of  typical atrial flutter, normally the femoral vein access is chosen. In case of obstruction of the IVC, the access via SVC is most common.  In literature there are only few reports about similar situations. Authors referred about cases, where the access to successful CTI ablation was chosen via the jugular access due to total cavopulmonal connection [6], an interruption [7] or absence [8-10] of the IVC. Guenther et al. described a case where ablation of CTI was performed through the azygos vein because of absence of the perihepatic IVC.

The obstacle to the right atrium in our patient with typical flutter  was an implanted filter in the IVC for secondary prevention of pulmonary embolism which made us choose the jugular / subclavian access. The inferior access via femoral vein allows ablation of CTI by pulling the maximally angulated ablation catheter from the ventricular aspect to the edge of IVC and thus generate a certain pressure on it, leading to better contact and energy delivery.

Using  the 'superior' access the catheter has to be pushed instead of being pulled against the CTI. This leads to extreme instability and finally makes the catheter fall down into IVC without ablating the important transition zone between CTI and IVC. The manoeuvre that helped us to resolve this problem after completion of the first part of ablation as described was the turn of the catheter 'upside down' making the tip look caudal towards IVC angulated against CTI. From that point we started the second 'semi-line' moving the catheter upwards finally connecting the two lines.This allowed us to apply a certain push upon the atrial aspect of CTI, gain best possible stability and finally succeed to reach a bidirectional block. In cases of typical flutter ablation with no access via IVC, the jugular / subclavian access via SVC using the described manoeuvre is a practicable and safe approach to achieve bidirectional isthmus block.
